# Identification of Novel Mutations in STAR Gene in Patients with Lipoid Congenital Adrenal Hyperplasia: A First Report from India

**DOI:** 10.4274/Jcrpe.927

**Published:** 2013-05-30

**Authors:** Lakshmi Vasudevan, Rajesh Joshi, Dhanjit Kumar Das, Sudha Rao, Daksha Sanghavi, Shiny Babu, Parag M. Tamhankar

**Affiliations:** 1 Genetic Research Center, National Institute for Research in Reproductive Health, JM Street, Parel, Mumbai; 2 Bai Jerbai Wadia Hospital for Children, Department of Pediatrics, Division of Pediatric Endocrinology, Parel, Mumbai

**Keywords:** Lipoid congenital adrenal hyperplasia, STAR gene, mutation, XY sex reversal

## Abstract

Lipoid congenital adrenal hyperplasia (LCAH), a rare disorder of steroid biosynthesis, is the most severe form of CAH. We report novel molecular findings of three unrelated infants with LCAH diagnosed at our center. A known missense mutation c.653C>T (p.A218V) and two novel mutations [premature termination c.441G>A (or p.W147X) and frameshift deletion c.del815G (or p.R272PfsX35)] were identified after complete sequencing of the STAR gene. Prenatal diagnosis was carried out for the family with mutation c.815delG by molecular testing wherein the fetus was found to be homozygous for the mutation. This is the first report of molecular diagnosis and prenatal testing for LCAH from India.

**Conflict of interest:**None declared.

## INTRODUCTION

Lipoid congenital adrenal hyperplasia (LCAH) is an inherited genetic disorder of adrenal and gonadal steroidogenesis. Most cases are caused by mutations in steroid acute regulatory (STAR) gene (MIM 600617, locus 8p11.23) which encodes a protein responsible for transport of cholesterol from outer to inner mitochondrial membrane (1). In few cases, mutations in the CYP11A1 (MIM 118485, locus 15q24.1) gene encoding the cytochrome P450 cleavage enzyme have also been reported to cause LCAH (2). The inheritance is autosomal recessive. The affected children present with signs of adrenal failure early in life and additionally male children with 46,XY chromosomal complement are reared as females due to absence of virilization of external genitalia. In this paper, we report novel mutations in the STAR gene in three unrelated infants with LCAH diagnosed at our center. Prenatal diagnosis was carried out for one family by molecular testing and the fetus was found to be homozygous for the mutation. This is the first report of molecular diagnosis of LCAH from India.

## CASE REPORTS

**Case 1:** A ten-month-old child reared as female was referred to our center for genetic consultation and diagnosis of 46,XY sex reversal and early-onset adrenal failure. She is the first born child to third-degree consanguineous parents and had presented at 45 days of life with generalized edema and tonic and clonic convulsions. On admission, the infant did not demonstrate any physical dysmorphisms. She had normal female external genitalia and the gonads were not palpable.A random plasma glucose was 71.7 mg%, serum electrolytes revealed low sodium (110 mEq/L), low chloride (90 mEq/L) and high potassium (6.7 mEq/L). Results of baseline serum hormonal analysis are given in Table 1. No substantial increase in levels of hormones was found following 250 mg synacthen test (DHEA was 3.18 mg /mL and androstenedione was less than 0.3 mg /mL). She was treated with intravenous saline, glucocorticoids and mineralocorticoids for adrenal insufficiency. A computed tomography (CT) scan of the abdomen showed fatty deposits in both adrenal fossae with non-visualization of the adrenal glands. Both testes were found in the inguinal region, and there were no Mullerian structures. The patient showed an adequate growth and development with this treatment.

**Case 2:** This patient was a 2.5-month-old female child, second born, of consanguineous parents. She was referred to our center for 46,XY sex reversal, adrenal insufficiency, and poor weight gain. She had presented with complaints of vomiting in the past 15 days and increased pigmentation of skin that was noticed from birth. On admission, the patient was dehydrated. She had normal female genitalia but increased generalized skin pigmentation. Investigations revealed hyponatremia (115 mEq/L) and hyperkalemia (6.5 mEq/L). Results of baseline serum hormonal analysis are given in Table 1. Baseline plasma renin activity was 35.5 ng/mL/hr (normal 4 to 8), while the aldosterone level was 98.9 pg/mL (normal 740 – 890). Synacthen test revealed the levels of cortisol to be less than 1 mg/dL at 30 min and also at 60 min. CT of the abdomen showed presence of testes in the inguinal region and nodular hyperplasia of the adrenals. Mullerian structures were not visualized. The patient was treated with intravenous saline, glucocorticoids, and mineralocorticoids for adrenal insufficiency.

**Case 3:** This patient was a 2.5–month-old infant, third born, of third-degree consanguineous parents and was referred to our center with 46,XY sex reversal and adrenal insufficiency. She was noted to have hyperpigmentation of skin in the face, trunk, palms, and soles. During her neonatal period, she had been admitted to a hospital for hyponatremic dehydration and convulsions. On admission, external genitalia examination revealed a normal clitoris, vulval hyperpigmentation, normal vaginal opening. The gonads were palpable in the vulvar region. Ultrasonography of the pelvis and inguinal region revealed presence of testes and related structures (right testis 1 x 0.8 cm; left 1.2 x 0.5 cm in diameter). Uterus was absent. Results of baseline serum hormonal analysis are given in Table 1.

## METHODS

Informed consent was obtained from all three families, and two mL blood was obtained from the patients and their parents. Genomic DNA was extracted using Qiagen Midi Kit following the manufacturer’s instructions. Polymerase chain reaction for all the exons and exon-intron boundaries of the STAR gene was done as described previously (1). DNA amplification was performed for each fragment in a 10 mL final volume containing 100 ng genomic DNA, 1 mM dNTPs, 10 pmol of each primer, 1 U Taq polymerase, and 1 mL 10 X PCR buffer (Kapa Biosystems, MA, USA). Thirty cycles of amplification were used, each consisting 1 min denaturation at 94°C, 45 s annealing at 60-65°C suitable for each exons, and 45 s extension at 72°C in a thermal cycler. Final extension time was 10 minutes. Negative control PCR tubes contained all of the above ingredients except DNA. PCR products were then electrophoresed in 2% agarose along with the appropriate negative controls and a 100 base-pair DNA ladder. Products that passed this quality check were purified by treatment with Exo-SAP-IT™ (USB Corporation, OH, USA) and then sequenced using BigDye Terminator v3.1; capillary electrophoresis was performed using an automated sequencer ABI-3730XL (Applied Biosystems, CA, USA). The patient sequences were compared to wild type using online BLAST (http://blast.ncbi.nlm.nih.gov/Blast.cgi). Pathogenicity of variants was determined using MutationTaster software (http://www.mutationtaster.org/). Mutations were described according to mutation nomenclature, considering nucleotide +1 the A of the first ATG translation initiation codon. Nucleotide numbers derived are cDNA STAR sequence RefSeq cDNA: NM_000349.2. Mutations found in patients were confirmed to be heterozygous in carrier parents. The mutations identified were then looked up in public databases like The Human Gene Mutation Database (http://www.hgmd.cf.ac.uk). 

## RESULTS

**Figure 1:**
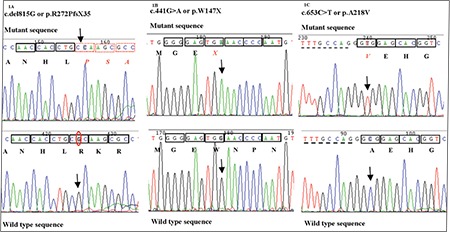
Sequence chromatograms of the mutations seen in our patients. The codons have been bracketed and amino acids have been placed in a text box below. The arrows indicate the site of mutation, dotted boxes indicate frameshift, and mutated amino acid

The proband in case 1 was found to have novel homozygous deletion of a single nucleotide G in exon 7 of the STAR gene. This mutation c.del815G leads to frameshift mutation p.R272PfsX35. The mutation was predicted to lead to elongation of the peptide chain by 34 amino acids. The proband in case 2 was found to be homozygous for the novel nucleotide substitution in exon 4 of the STAR gene c.441 G>A leading to the premature termination mutation p.W147X. The child’s parents were confirmed to be heterozygous for this mutation. The proband in case 3 showed homozygosity for a known missense mutation c.653C>T (p.A218V) in exon 6 of the STAR gene. Figure 1 shows chromatograms of all mutations. The mutations p.R272PfsX35 and p.W147X were determined pathogenic by MutationTaster software (probability of pathogenicity being 0.99 and 1, respectively). Carrier status of the above mutations was confirmed in the respective parents. Prenatal diagnosis in a subsequent pregnancy of the mother of case 1 revealed the fetus to be homozygous for the same mutation. The couple accepted medical termination of pregnancy.

## DISCUSSION

Classical type of LCAH was considered as the clinical diagnosis owing to a combination of infantile-onset adrenal insufficiency and complete sex reversal in these 46,XY infants. The clinical differentials of sex reversal with adrenal insufficiency include P450scc deficiency due to mutations in CYP11A1 gene, Smith-Lemli-Opitz syndrome (DHCR7 gene), NR5A1 (SF1) mutation-related sex reversal, CYP17 and 3-beta hydroxysteroid dehydrogenase deficiency (HSD3B2 gene) (3). Complete sex reversal in 46,XY males without adrenal insufficiency can also occur in conditions such as Leydig cell hypoplasia or due to mutations in SRY, DHH, SOX9, DMRT1, WNT1, or ATRX. Duplications in DAX1 ,WNT4, or RSPO1 also lead to sex reversal. Mutations in HSD17B3, SRD5A2, or AR cause defects in testosterone synthesis leading to sex reversal not associated with adrenal failure (3). The diagnosis of LCAH must be distinguished from other combined glucocorticoid and mineralocorticoid deficiencies. The distinction between LCAH and 21-hydroxylase deficiency is that the patients with LCAH have female external genitalia regardless of karyotype and very low or immeasurable levels of all steroid hormones, whereas the patients with 21-hydroxylase deficiency have high concentrations of 21-deoxysteroids, especially 17-hydroxyprogesterone, and affected 46,XX individuals are virilized (3). However, LCAH can be mistaken for 3b-hydroxysteroid dehydrogenase deficiency when 17- hydroxypregnenolone (which is grossly elevated in 3b-hydroxysteroid dehydrogenase deficiency) is not measured. The most difficult differential diagnosis probably is between LCAH and congenital adrenal hypoplasia. In the presence of female external genitalia and XY karyotype, lipid deposits in adrenals noted in the abdominal CT scan, as in our patients, strongly suggest LCAH. On the other hand, the diagnosis of LCAH in 46,XX patients generally depends on the radiographic demonstration of massively enlarged adrenals that are not found in adrenal hypoplasia (4). Pathological mutations found in the STAR gene confirmed the diagnosis of LCAH in our patients.

The LCAH phenotype is the result of two separate events (5). The genetic loss of steroid genesis resulting from mutation in the STAR gene is the primary defect; there is a subsequent loss of steroid genesis that is independent of STAR and which is due to cellular damage from accumulated cholesterol esters in the adrenal cortex, leading to salt wasting, hyponatremia, hypervolemia, hyperkalemia, hyperglycemia, acidosis, and death in infancy, although patients can survive to adulthood with appropriate mineralocorticoid and glucocorticoid-replacement therapy. Two of our patients demonstrated progressive decrease in the cortisol levels after birth (case 1 and case 3).

LCAH is caused by homozygous or compound heterozygous mutation in the gene encoding the steroidogenic acute regulatory protein (STAR; 600617) on chromosome 8p11.23. Classical type of LCAH is the most severe form of CAH in which the adrenals and gonads exhibit a severe defect in the conversion of cholesterol to pregnenolone. LCAH is common among the Japanese, Korean, and Palestinian Arab populations, but is rare elsewhere (5,6). Metherell et al (7) have previously found STAR gene mutation R188C in an Indian child in the UK who presented with familial glucocorticoid deficiency phenotype. However, no case has been reported to date from India.

Hyperpigmentation in cases of LCAH occurs due to hypersecretion of ACTH and was also observed in our cases. Hypoglycemia can occur due to glucocorticoid deficiency, but was not documented in our patients (8).

The novel mutations p.R272PfsX35 and p.W147X in our cases lead to premature termination of the translation. According to the 50–54-nt boundary rule, the mutation p.R272PfsX35 would be translated into abnormal polypeptide chain, but the mutation p.W147X would lead to nonsense mediated decay of the mRNA (9). The mutation would affect the START domain of the StAR protein which includes 214 amino acids (from 67 to 280) (10). The StAR protein plays a key role in steroid hormone synthesis by mediating the transfer of cholesterol from the outer mitochondrial membrane to the inner mitochondrial membrane where it is cleaved to pregnenolone. The START domain binds lipids and the C terminal of the domain is crucial to form a lid over the lipid-binding pocket that shields the ligand (lipid or sterol) from the external environment (11). The A218V mutation was previously identified in patients from Japan and was demonstrated to lead to formation of protein that reaches the inner mitochondrial membrane but is functionally inactive (8).

Affected individuals usually have a severe deficiency of adrenal and gonadal steroids. All affected individuals are phenotypic females irrespective of gonadal sex, and frequently die in infancy if mineralocorticoids and glucocorticoids are not replaced (5). All our patients were treated with mineralocorticoid and glucocorticoid replacement. On follow-up, they are showing normal growth and development.

Prenatal diagnosis is important to enable couples to exercise their reproductive choice (12). In our series, all couples received genetic counseling and were informed about the possibility of prenatal diagnosis in their future pregnancies. Prenatal diagnosis followed by medical termination of pregnancy was found acceptable in this small series.

## CONCLUSION

LCAH should be considered an important possibility in the differential diagnosis for neonatal-/ infantile-onset adrenal insufficiency. Mutation analysis of the STAR gene is essential for definitive diagnosis, genetic counseling, and prenatal diagnosis of LCAH. 

## Figures and Tables

**Table 1:**
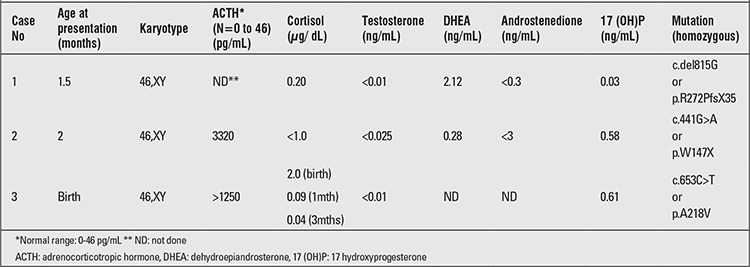
Hormonal profile and results of genetic testing in patients with lipoid congenital adrenal hyperplasia
